# Supplementary material for: Prevalence of depressive symptoms among secondary school adolescents in Dodoma and Pwani, Tanzania
Source: PLOS Ment Health. 2026 May 29;3(5):e0000518. doi: 10.1371/journal.pmen.0000518 (PMC13221033; doi:10.1371/journal.pmen.0000518)
Supplement: Attachment — Submitted filename: RESPONSE TO REVIEWERS letter.docx [file pmen.0000518.s002.docx]

**RESPONSE TO REVIEWERS**

**Manuscript Title:** *Prevalence of Depressive Symptoms Among Adolescents in Secondary Schools in Dodoma and Pwani, Tanzania*
**Manuscript ID:** PMEN-D-25-00519

Dear Editor and Reviewers,

We sincerely thank the Editor and the reviewers for their valuable comments and constructive suggestions. We have carefully revised the manuscript accordingly, and we believe the changes have significantly improved its clarity, methodological rigour, and overall quality. Below, we provide a point-by-point response to each comment.

Reviewer Comment 1: The title should reflect depressive symptoms rather than depression.

**Response:**
Thank you for this important observation. The title has been revised to ensure conceptual accuracy and alignment with the study's measurement tool. The revised title now reads: *“Prevalence and of Depressive Symptoms Among Adolescents in Secondary Schools in Dodoma and Pwani, Tanzania.”* The term “depressive symptoms” has also been used consistently throughout the manuscript.

Reviewer Comment 2: The introduction contains information that should be in the problem statement; avoid repetition.

**Response:**
We appreciate this suggestion. The manuscript has been restructured to clearly distinguish between the Introduction and the Statement of the Problem. Specifically, detailed Tanzania-specific statistics and empirical evidence were moved to the problem statement section, while the Introduction now provides a broader global and regional context and highlights the research gap. Redundant information has been removed to improve clarity and flow.

Reviewer Comment 3: The study design section should only describe the design; move tools and analysis elsewhere.

**Response:**
Thank you for this clarification. The Study Design section has been revised to focus exclusively on the cross-sectional design and its justification. Information regarding the data collection instrument (PHQ-9) and statistical analysis procedures has been relocated to the Data Collection and Data Analysis sections, respectively.

Reviewer Comment 4: Clarify the term “non-clinical mental health status.”

**Response:**
We thank the reviewer for highlighting this ambiguity. The phrase “non-clinical mental health status” has been removed, and the text has been revised to clearly state that participants reported “no depressive symptoms,” consistent with PHQ-9 classification.

Reviewer Comment 5: Figures are missing.

**Response:**
Thank you for noting this. The relevant figure has now been prepared and submitted as a separate file in accordance with the journal’s submission guidelines. The manuscript text has been revised to appropriately refer to the figure (Figure 1).

Reviewer Comment 6: Use “depressive symptoms” instead of “depression symptoms.”

**Response:**
We appreciate this important correction. The term “depressive symptoms” has been used consistently throughout the manuscript, including in tables, figures, and text.

Reviewer Comment 7: Comparison with other studies should be integrated into the discussion; avoid repetition of results.

**Response:**
Thank you for this valuable suggestion. The discussion section has been substantially revised. The separate “Comparison with other studies” section has been removed, and comparisons have been integrated within the interpretation of findings. Repetition of results has been minimised, and the discussion now focuses on explanation, contextualization, and implications.

Reviewer Comment 8: Clarify consent procedures for different age categories.

**Response:**
We appreciate this important ethical observation. The ethical section has been revised to clearly specify that participants below 18 years provided assent alongside parental/guardian consent (facilitated through school administration), while participants aged 18 years and above provided their own informed consent. This clarification ensures alignment with ethical standards.

Editor Comment: Data availability and repository deposition**.**

**Response:**
Thank you for this guidance. We are in the process of depositing the anonymised dataset into the appropriate repository. The dataset will be made available in accordance with the journal’s open data policy while ensuring participant confidentiality. The data availability statement has been updated accordingly.

We hope that the revisions satisfactorily address all concerns. We are grateful for the insightful feedback, which has significantly improved the manuscript.

Yours sincerely,
**Divine Patrick Mwaluli**
